# Preferências dos Pacientes após Estreitamento Coronário Recorrente: Experimentos de Escolha Discreta

**DOI:** 10.36660/abc.20190305

**Published:** 2020-10-13

**Authors:** Carlos Alberto da Silva Magliano, Andrea Liborio Monteiro, Amanda Rebeca de Oliveira Rebelo, Giovanna Francisconi Santos, Claudia Cristina de Aguiar Pereira, Nikolas Krucien, Roberto Magalhães Saraiva

**Affiliations:** 1 Instituto Nacional de Cardiologia Rio de JaneiroRJ Brasil Instituto Nacional de Cardiologia, Rio de Janeiro, RJ – Brasil; 2 University of Illinois at Chicago ChicagoIllinois EUA University of Illinois at Chicago, Chicago, Illinois - EUA; 3 Fundação Oswaldo Cruz Escola Nacional de Saúde Pública Rio de JaneiroRJ Brasil Escola Nacional de Saúde Pública, Fundação Oswaldo Cruz, Rio de Janeiro, RJ - Brasil; 4 University of Aberdeen Aberdeen Reino Unido University of Aberdeen, Aberdeen - Reino Unido; 5 Fundação Oswaldo Cruz Instituto Nacional de Infectologia Evandro Chagas Rio de JaneiroRJ Brasil Instituto Nacional de Infectologia Evandro Chagas, Fundação Oswaldo Cruz, Rio de Janeiro, RJ - Brasil

**Keywords:** Doença da Artéria Coronariana/cirurgia, Revascularização Miocárdica, Intervenção Coronária Percutânea, Angioplastia, Reestenose Coronária, Preferência do Paciente, Inquéritos e Questionários

## Abstract

**Fundamento:**

Selecionar a estratégia de tratamento ideal para a revascularização coronária é um desafio. Um desfecho crucial a ser considerado no momento dessa escolha é a necessidade de refazer a revascularização, uma vez que ela se torna muito mais frequente após a intervenção coronária percutânea (ICP) do que após a cirurgia de revascularização do miocárdio (CRM).

**Objetivo:**

Pretende-se, com este estudo, trazer reflexões acerca das preferências dos pacientes pelas estratégias de revascularização sob a perspectiva de pacientes que tiveram que refazer a revascularização.

**Métodos:**

Selecionamos uma amostra de pacientes que haviam sido submetidos à ICP e hospitalizados para refazer a revascularização coronária e elicitamos suas preferências por nova ICP ou CRM. Morte perioperatória, mortalidade a longo prazo, infarto do miocárdio e repetir a revascularização foram utilizados para a construção de cenários a partir da descrição de tratamentos hipotéticos que foram rotulados como ICP ou CRM. A ICP era sempre apresentada como a opção com menor incidência de morte perioperatória e maior necessidade de se refazer o procedimento. O modelo logístico condicional foi empregado para analisar as escolhas dos pacientes, utilizando-se o software R. Valores de p <0,05 foram considerados estatisticamente significativos.

**Resultados:**

Ao todo, 144 pacientes participaram, a maioria dos quais (73,7%) preferiram a CRM à ICP (p < 0,001). Os coeficientes de regressão foram estatisticamente significativos para o rótulo ICP, mortalidade a longo prazo da ICP, morte perioperatória da CRM, mortalidade a longo prazo da CRM e refazer a CRM. O rótulo ICP foi o parâmetro mais importante (p < 0,05).

**Conclusão:**

A maioria dos pacientes que enfrentam a necessidade de refazer a revascularização coronária rejeitam uma nova ICP, com base em níveis realistas de riscos e benefícios. Incorporar as preferências dos pacientes à estimativa do risco-benefício e às recomendações de tratamento poderia melhorar o cuidado centrado no paciente.

## Introdução

A doença cardíaca coronária é a principal causa de mortalidade e incapacidade no mundo, responsável por cerca de um terço de todas as mortes em indivíduos acima de 35 anos.^1^ Há duas opções de revascularização: a intervenção coronária percutânea (ICP) e a cirurgia de revascularização do miocárdio (CRM). Além da necessidade de a CRM ser realizada com o peito aberto, algumas diferenças cruciais entre os tratamentos são: o risco de morte perioperatória, mais elevado na CRM e o risco de ter que refazer a revascularização, mais elevado na ICP.^2^ Recentemente, o uso de *stents* recobertos reduziu a necessidade de refazer a revascularização, mas o dilema em relação à melhor estratégia de revascularização permanece sem resposta.^3^^,^^4^ Portanto, a escolha da estratégia de revascularização ideal é um desafio e depende de muitos fatores, tais como o número, a gravidade, e a posição das artérias com estreitamento ou bloqueadas, a saúde geral do paciente, e suas preferências pelos desfechos relacionados, como o tempo de recuperação, as complicações em curto prazo, a necessidade de refazer a revascularização e a sobrevida a longo prazo.^5^

Os prestadores de cuidados de saúde vêm tentando integrar os pacientes de forma mais ativa nas decisões e o profissional deve possuir as habilidades para envolver os pacientes nas tomadas de decisão.^6^ Simplesmente pedir aos pacientes para ranquear os desfechos relacionados ao tratamento, de modo geral, não produz informações substanciais, uma vez que eles provavelmente irão afirmar que almejam todos os benefícios (baixos riscos para todos os desfechos). Por outro lado, os experimentos de escolha, como os experimentos de escolha discreta (DCE) obrigam os pacientes a fazer um *trade-off* entre opções realistas, por exemplo, a opção com o menor risco de morte perioperatória (ICP) *versus* a opção com menor risco de ter que refazer a revascularização (CRM).

Os DCEs são frequentemente utilizados para obtenção de preferências numa grande variedade de situações e se tornaram a abordagem aplicada com mais frequência nos cuidados de saúde.^7^ Em um DCE, uma sequência de cenários hipotéticos é apresentada aos indivíduos e pede-se que eles escolham entre alternativas concorrentes, que variam quanto a diversas características (atributos).

A metodologia do DCE é fundamentada em um modelo de maximização da utilidade aleatória (*Random Utility Maximization-RUM*), onde as premissas básicas são: 1) qualquer produto, nesse caso a opção de tratamento (ICP e CRM) pode ser caracterizado por atributos-chave (por exemplo, risco de morte perioperatória, risco de refazer a revascularização) e seus níveis (por exemplo, 2%, 35%) e 2) sempre que os indivíduos têm opções dentre as quais escolher (por exemplo, PCI versus CABG), eles escolhem a opção que tem a maior utilidade, que é definida através da comparação dos níveis daqueles atributos.^8^ A utilidade é um termo utilizado pelos economistas para descrever a mensuração da “usabilidade” e “desejabilidade” que um consumidor obtém a partir de qualquer bem e representa a capacidade de um produto trazer satisfação.

Em uma revisão sistemática publicada recentemente, nosso grupo de pesquisa procurou estudos que avaliassem preferências declaradas entre ICP e CRM. Identificamos uma escassez de estudos que abordassem esse tema e uma falta de métodos padronizados para avaliar as preferências dos pacientes. Ainda assim, quatorze desfechos utilizados para comparar ICP e CRM puderam ser identificados: fibrilação atrial, insuficiência cardíaca, cicatriz de incisão, tempo de internação, mortalidade a longo prazo, infarto do miocárdio, morte perioperatória, infecção pós-operatória, angina pós-procedimento, pseudoaneurisma, insuficiência renal, refazer CRM, refazer ICP, e acidente vascular cerebral (AVC).^9^

Dentre aqueles que já haviam sido submetidos à ICP, não há nenhum estudo que avalie as preferências dos pacientes entre se submeter a nova ICP ou CRM, caso nova revascularização seja indicada. Portanto, o objetivo deste estudo foi trazer reflexões acerca das preferências dos pacientes pela ICP ou CRM sob a perspectiva de pacientes hospitalizados que tiveram que refazer a revascularização.

## Métodos

### Desenho

Um DCE foi desenvolvido e administrado em uma amostra de pacientes hospitalizados utilizando-se entrevistas presenciais individuais, entre novembro de 2017 e abril de 2018. Os pacientes foram recrutados aleatoriamente com base no número do leito, usando uma lista de números aleatórios no Instituto Nacional de Cardiologia, um hospital público terciário brasileiro especializado em Cardiologia. Pacientes com 18 anos de idade ou mais foram considerados elegíveis se tivessem sido submetidos à ICP prévia e estivessem internados devido à doença coronária, necessitando de nova revascularização.

Os pacientes que se consideraram incapazes de entender o experimento foram excluídos. Não houve nenhum outro critério de exclusão. Foi obtida a aprovação ética do Comitê Ético do Instituto Nacional de Cardiologia e um consentimento informado por escrito foi obtido de cada participante do estudo (CAAE número 63684017.0.0000.5240).

### Experimento de Escolha Discreta

O DCE baseou-se nos desfechos que foram identificados na revisão sistemática publicada anteriormente.^9^ Para realizar o experimento de escolha discreta, aqueles desfechos foram previamente ranqueados e receberam pontos dos pacientes para identificar sua importância relativa. Todos os desfechos foram ranqueados levando-se em consideração um cenário hipotético. O método detalhado utilizado para os pacientes ranquearem e distribuírem pontos entre os desfechos foi publicado anteriormente.^10^ A seleção de quais atributos devem ser utilizados nos cenários do DCE é uma etapa essencial, uma vez que só será possível calcular os *trade-offs* entre os atributos que serão utilizados. Incluímos somente quatro atributos, já que a utilização de todos os 14 atributos identificados na revisão sistemática faria com que os entrevistados ficassem cansados ou fizessem uso de heurísticas, um atalho mental que permite que as pessoas façam julgamentos rapidamente, embora resulte em medidas de preferência enviesadas.^11^ Os quatro atributos escolhidos para compor os cenários do DCE foram selecionados considerando-se: 1) a mortalidade a longo prazo deveria ser incluída como a referência para taxas marginais de substituição; 2) serem os atributos mais relevantes, conforme o ranqueamento feito pelos pacientes, e 3) terem uma diferença significativa na incidência entre ICP e CRM. Os quatro atributos selecionados foram: morte perioperatória, mortalidade a longo prazo, infarto do miocárdio e refazer a revascularização.

Para aplicar o DCE em pacientes hospitalizados, utilizamos recursos visuais que foram especialmente desenvolvidos para esse projeto, a fim de incluir pacientes com diferentes origens socioeducativas.^12^ Os recursos visuais melhoram a compreensão do risco e permitem que os pacientes se considerem aptos a entender e participar das decisões com respostas consistentes com a teoria econômica, escolhendo as alternativas com maior utilidade.

**Figura 1:**
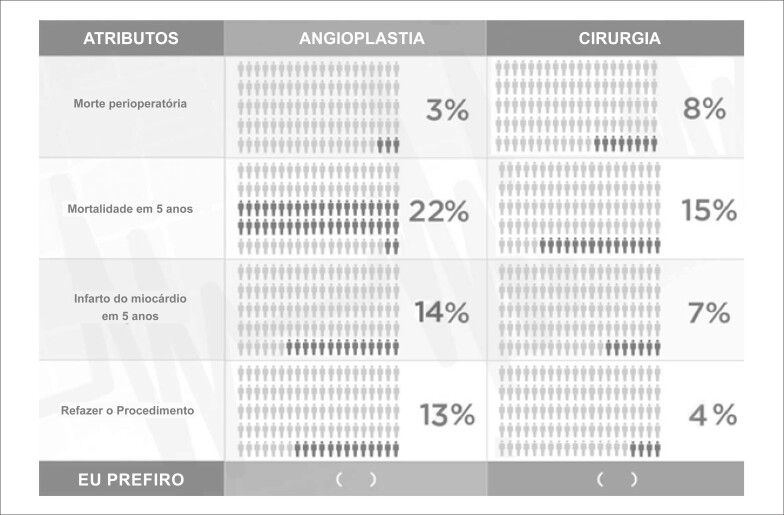
Exemplos de opções para um experimento de escolha discreta.

Um exemplo de cenário de DCE apresentado neste artigo aos pacientes encontra-se na Figura 1: o primeiro atributo, “morte perioperatória”, aparece com nível 3% para ICP (angioplastia) e 8% para CRM (cirurgia); o segundo atributo é “mortalidade em 5 anos”, risco de 22% para ICP e 15% para CRM. Cada entrevistado teve que escolher entre ICP e CRM em 12 cenários diferentes. Todos os cenários utilizados foram apresentados com os mesmos quatro atributos, mas com diferentes níveis de combinação, de acordo com valores preestabelecidos. A ICP sempre foi apresentada como a opção com menor risco de morte perioperatória e maior necessidade de refazer o procedimento (Tabela 1).

**Tabela 1:** Atributos e níveis selecionados para descrever as opções de tratamento no DCE

Atributo	ICP	CRM
Morte perioperatória	1% - 2% - 3%	4% - 6% - 8%
Mortalidade a longo prazo	8% - 15% - 22%	7% - 11% - 15%
Infarto do miocárdio	6% - 10% - 14%	3% - 5% - 7%
Repetir a revascularização	13% - 24% - 35%	1% - 4% - 7%

CRM: cirurgia de revascularização do miocárdio; ICP: intervenção coronária percutânea.

### Desenvolvimento da Pesquisa DCE – Seleção de Níveis

Quando as opções de tratamento foram descritas nas tarefas do DCE, os quatro atributos de risco foram operacionalizados através da sua classificação em três níveis específicos. Os níveis de mortalidade a longo prazo, revascularização, e infarto do miocárdio foram obtidos de estudos recentes comparando ICP e CRM,^4^^,^^13^^–^^17^ para garantir que níveis reais de risco seriam utilizados. O nível para morte perioperatória foi selecionado com base na média da morte perioperatória da ICP e da CRM (2,21% e 6,23%, respectivamente), de acordo com os dados do DATASUS, anos 2016 e 2017,^18^ e foram apresentados em três níveis: 1%, 2% ou 3% para ICP e 4%, 6% e 8% para CRM (Tabela 1).

### Desenvolvimento da Pesquisa DCE – Desenho das Tarefas de Escolha

O programa NGene^19^ foi utilizado para projeção dos cenários, o que correspondeu ao mecanismo pelo qual os perfis hipotéticos foram apresentados aos entrevistados para obtenção das preferências no DCE.^11^ Um desenho D-Eficiente, sem nenhuma informação prévia sobre as preferências dos pacientes, foi utilizado para gerar as tarefas de escolha. A ordem das tarefas de escolha foi randomizada entre os participantes.

Todos os pacientes foram entrevistados individual e presencialmente, escolhendo uma opção em 12 cenários diferentes apresentados em um questionário impresso.

### Análise Estatística

A regressão logística condicional foi empregada para analisar as escolhas dos pacientes, utilizando-se o software R. Os dados de medição foram apresentados como média ± desvio padrão (x ± DP). Valores de p <0,05 foram considerados estatisticamente significativos.

Os quatro atributos entraram no modelo como variáveis contínuas e lineares. Uma vez que as preferências dos pacientes pelos atributos de risco foram estimadas, foi possível computar taxas marginais de substituição (TMS). A TMS representou os *trade-offs* entre os atributos ou o quanto de um atributo os pacientes estavam dispostos a sacrificar para obter mais de um outro atributo. Devido à especificação linear do modelo, a TMS consistiu simplesmente da razão de dois coeficientes estimados.^20^ Seguimos essa abordagem para computar os Riscos Máximos Aceitáveis com um aumento de 1% na mortalidade a longo prazo na revascularização como referência.

## Resultados

Do total de 145 pacientes recrutados, 144 entregaram o consentimento informado por escrito para participar do estudo e se consideraram capazes de entender o experimento. A idade média foi de 57,5 ± 11,6 anos; 74% eram homens e a maioria dos pacientes eram casados (56%), com baixos níveis de escolaridade e renda (Tabela 2).

**Tabela 2:** Condições socioeconômicas de base e características dos entrevistados

Característica	Dado (N = 144)
Idade, anos	57,5 (11,6)
Sexo masculino, número (%)	106 (74%)
Renda anual, U$	6.838,59 (10.586,82)*
	Casado 81 (56%)
Estado Civil	Solteiro 35 (24%)
	Outro 28 (20%)
	≤ 1 ano: 5 (3,0%)
	2 – 5 anos: 39 (27%)
Nível de escolaridade, anos de estudo (%)	6 – 9 anos: 31 (22%)
	10 – 12 anos: 40 (28%)
	Nível Superior: 29 (20%)
	1 – 98 (68%)
Número de ICPs prévias	2 – 23 (16%)
	3 ou mais – 23 (16%)

ICP: intervenção coronária percutânea; dados contínuos são apresentados como média (desvio padrão).

* conversão com base em http://www4.bcbgov.br/pec/conversao/conversao.asp (1 USD=3,49 BRL).

Cada entrevistado respondeu a 12 tarefas de escolha, totalizando, assim, 1.728 (ou seja, 144 vezes 12) observações para análise. A maioria dos pacientes (73,7%) preferiu a CRM à ICP (p < 0,001). Os resultados para a estimativa das preferências estão apresentados na Tabela 3.

**Tabela 3:** Pesos Relativos das Preferências Estimadas

Parâmetro	Estimativa	Erro Padrão	Valor de p
Rótulo ICP	- 1,3226	0,6708	< 0,05
Morte perioperatória da ICP	- 0,0421	0,0975	NS
Mortalidade a longo prazo da ICP	- 0,0371	0,0172	< 0,05
Infarto do miocárdio da ICP	- 0,0314	0,0165	NS
Repetir ICP	- 0,0005	0,0087	NS
Morte perioperatória da CRM	- 0,0956	0,0425	< 0,05
Mortalidade a longo prazo da CRM	- 0,0582	0,0287	< 0,05
Infarto do miocárdio da CRM	0,0480	0,0407	NS
Repetir CRM	- 0,0657	0,0253	< 0,05

Verosimilhança logarítmica = −952,35. CRM: cirurgia de revascularização do miocárdio; ICP: intervenção coronária percutânea; NS: não significante.

Os coeficientes de regressão foram estatisticamente significativos no nível de 5% para o rótulo ICP, mortalidade a longo prazo da ICP, morte perioperatória da CRM, mortalidade a longo prazo da CRM e refazer a CRM. Os coeficientes negativos indicam que os pacientes consideraram os atributos como algo indesejável (mais risco é pior do que menos). Curiosamente, a função utilidade usada no modelo de regressão incluiu uma constante alternativa específica para o rótulo ICP e foi não apenas estatisticamente significativa, como também o parâmetro mais importante, aquele com o maior valor negativo, o que significa que a maioria dos pacientes que tiveram que refazer a revascularização rejeitaram a ICP, independentemente dos riscos associados apresentados.

## Discussão

O presente estudo é único porque, até onde sabemos, é o primeiro a avaliar as preferências dos pacientes dentre aqueles que tiveram que refazer a revascularização após a ICP e fornece importantes reflexões, tais como a evidência de uma variação significativa na utilidade percebida de tratamentos e a preferência notória, de modo geral, pela opção mais invasiva (CRM).

Poucos estudos utilizaram o DCE como uma ferramenta para elicitação de preferências para revascularização coronária. Nossa revisão sistemática identificou que a maioria dos estudos (83%) utilizaram o ranqueamento e a pontuação como métodos para identificar as preferências dos pacientes e apenas dois estudos (33%)^21^^,^^22^ utilizaram cenários hipotéticos. Hornberger et al.^22^ analisaram uma amostra nacional de entrevistados em um estudo de análise conjunta considerando cicatriz da incisão, dor, tempo de recuperação, tempo de internação hospitalar e refazer o tratamento. Vale ressaltar que os participantes consideraram que a ICP superaria a CRM apenas se o risco de ter que refazer a revascularização ao longo de três anos fosse reduzido para menos de 28%. Kipp et al.,^21^ utilizando um modelo de regressão logística mista, identificaram que, para quase todos os riscos indicados, os pacientes preferiram a ICP à CRM, até mesmo quando o risco de morte era duas vezes maior que o risco da CRM ou o risco de refazer os procedimentos era três vezes superior quando comparado com a CRM.

Contrariamente ao estudo realizado por Kipp, a maioria dos pacientes (73,8%) neste estudo escolheu a opção mais invasiva: CRM. Essa diferença pode estar relacionada à população estudada, uma vez que levamos em consideração apenas pacientes com uma história prévia de ICP. Além disso, devemos considerar algumas diferenças nos desenhos dos estudos. Enquanto o estudo de Kipp et al.,^21^ baseou-se em um risco três vezes maior de refazer a ICP, quando comparado com o risco de refazer a CRM, com níveis entre 2 e 5%, nós consideramos o risco da CRM entre 1% e 7% e o da ICP entre 13% e 35%. Esse risco elevado de ter que refazer a ICP foi observado em pacientes diabéticos no estudo Syntax,^23^ no qual 35,3% dos pacientes acompanhados durante 5 anos tiveram que ser submetidos a um novo procedimento de revascularização.

Outro ponto importante levantado pelos nossos achados é que diferentes desfechos são vistos de forma diferente pelos pacientes. Entretanto, as recomendações das diretrizes se baseiam na utilização de desfechos combinados, tais como eventos cardiovasculares adversos importantes (MACE - *major adverse cardiovascular events*). Desfechos como morte, AVC, infarto do miocárdio e refazer a revascularização são frequentemente agrupados na tentativa de capturar o efeito global do tratamento, e as principais vantagens são a menor duração, tamanho da amostra e custo de um experimento clínico.^24^ O uso do MACE pressupõe que todos os seus componentes têm a mesma gravidade clínica e que os pacientes e médicos têm uma percepção semelhante de cada componente, hipóteses que se mostraram falsas tanto no nosso estudo quanto em outros.^24^^–^^26^ Pacientes e médicos têm perspectivas diferentes e nenhum deles considerou todos os desfechos clínicos da mesma forma. O peso apropriado de cada componente de um desfecho combinado forneceria uma interpretação mais precisa dos dados do experimento.

Uma importante decisão na aplicação do DCE é se as escolhas serão apresentadas como rotuladas ou não rotuladas. Decidimos adotar os cenários rotulados, ou seja, os pacientes escolhem entre ICP e CRM, e não a opção “A” *versus* “B”. Os DCEs não rotulados seriam mais indicados para investigar *trade-offs* entre atributos, ao passo que os DCEs rotulados podem ser mais adequados para explicar escolhas da vida real. Os conjuntos de escolha rotulados são considerados menos abstratos e podem aumentar a validade dos resultados, o que pode ser mais adequado para apoiar a tomada de decisões ao nível da definição de políticas. A desvantagem é que os rótulos podem diminuir a atenção dispensada pelos entrevistados aos atributos e alguns pacientes podem ter escolhido uma opção independentemente dos seus riscos.^27^ Na nossa amostra, cada entrevistado respondeu a uma tarefa adicional de escolha para determinar a validade do DCE ao final da seção: uma pergunta dominante, onde a ICP representava o tratamento com níveis de atributos claramente dominantes ou melhores, isto é, a opção menos invasiva associada a riscos mais baixos de mortalidade, infarto do miocárdio ou repetição do tratamento. Esperava-se que os entrevistados escolhessem a ICP, mas 54 (37,5%) dos pacientes escolheram a CRM, o que pode configurar rejeição prévia à ICP e o impacto da utilização do rótulo.

### Pontos Fortes e Implicações Clínicas

Há apenas alguns estudos sobre preferências dos pacientes entre a ICP e CRM e este é o primeiro a analisar as preferências dos pacientes especificamente por procedimentos de revascularização repetidos.

Outro ponto forte é a seleção de participantes, composta de pacientes hospitalizados, aguardando nova revascularização. Atualmente, a maioria dos valores dos estados de saúde são obtidos de membros do público em geral, que tentam imaginar como o estado seria, baseando-se sobretudo na premissa de que a saúde é paga pela população em geral. Entretanto, os pacientes compreendem melhor as consequências das suas escolhas e sabem como é viver com aquela condição de saúde. Isto minimiza uma das principais preocupações em relação ao DCE, que é o viés hipotético relacionado ao desinteresse ou desatenção pelos cenários hipotéticos, ao passo que os pacientes que enfrentam o problema de saúde estariam mais envolvidos no experimento.

As diretrizes atuais da Cardiologia podem se beneficiar da inclusão das preferências dos pacientes nas suas recomendações. Por exemplo, levando-se em consideração os resultados para os pacientes com doença de três vasos do estudo Syntax, a mortalidade a longo prazo de 11,4% no grupo CRM (valor do coeficiente – 0,0582) seria equivalente a uma mortalidade a longo prazo de 17,9% ((-0,0582/-0,0371)*11,4) no grupo ICP (valor do coeficiente −0,0371). Com base no valor dos parâmetros identificados no nosso modelo de regressão, mesmo com a mortalidade a longo prazo mais elevada para ICP (13,9%), essa diferença de 2,5% na mortalidade a longo prazo, observada no estudo Syntax, não seria suficiente para influenciar as preferências dos pacientes em favor da CRM.

### Limitações

Os resultados do nosso estudo são limitados pelo tamanho reduzido da amostra utilizada oriunda de um único hospital terciário, o que pode limitar a generalização dos nossos resultados.

Podem haver alguns efeitos de interação, uma vez que os pacientes podem ter valorado determinados atributos ou níveis de maneira diferente em decorrência das suas experiências particulares prévias. Outra questão é que os atributos entraram no modelo como variáveis contínuas para facilitar a compreensão, e consideramos o efeito das preferências dos níveis como algo linear, o que pode não ser realista, uma vez que o valor da mudança do risco baixo para moderado não é necessariamente o mesmo valor da mudança do risco moderado para grave.

## Conclusão

Apesar dos importantes *trade-offs* entre a ICP e CRM, tais como necessidade de refazer a revascularização, as preferências dos pacientes podem ter sido pouco exploradas. No DCE com uma amostra de pacientes hospitalizados com doença coronária e ICP prévia, nossos resultados sustentam que a maioria dos pacientes rejeita uma nova ICP e preferem a CRM, quando expostos aos níveis de risco reais de cada opção.
