# Supplementary material for: Preferências dos Pacientes após Estreitamento Coronário Recorrente: Experimentos de Escolha Discreta
Source: Arq Bras Cardiol. 2020 Oct 13;115(4):613–9. [Article in Portuguese] doi: 10.36660/abc.20190305 (PMC8386978; doi:10.36660/abc.20190305)
Supplement: Translation

# Translation: Patients' Preferences after Recurrent Coronary Narrowing: Discrete Choice Experiments

**Authors:** Carlos Alberto da Silva Magliano, Andrea Liborio Monteiro, Amanda Rebeca de Oliveira Rebelo, Giovanna Francisconi Santos, Claudia Cristina de Aguiar Pereira, Nikolas Krucien, Roberto Magalhães Saraiva

**Affiliations:** 1 Rio de JaneiroRJ Brazil Instituto Nacional de Cardiologia, Rio de Janeiro, RJ – Brazil; 2 ChicagoIllinois USA University of Illinois at Chicago, Chicago, Illinois - USA; 3 Rio de JaneiroRJ Brazil Escola Nacional de Saúde Pública, Fundação Oswaldo Cruz, Rio de Janeiro, RJ - Brazil; 4 Aberdeen United Kingdom University of Aberdeen, Aberdeen - United Kingdom; 5 Rio de JaneiroRJ Brazil Instituto Nacional de Infectologia Evandro Chagas, Fundação Oswaldo Cruz, Rio de Janeiro, RJ - Brazil

**Keywords:** Coronary Artery Disease/surgery, Myocardial Revascularization, Intervention Coronary Percutaneous, Coronary Restenosis, Patient Preference, Surveys and Questionnaires

## Abstract

**Background:**

Selecting the optimal treatment strategy for coronary revascularization is challenging. A crucial endpoint to be considered when making this choice is the necessity to repeat revascularization since it is much more frequent after percutaneous coronary intervention (PCI) than after coronary artery bypass grafting (CABG).

**Objective:**

This study intends to provide insights on patients' preferences for revascularization, strategies in the perspective of patients who had to repeat revascularization.

**Methods:**

We selected a sample of patients who had undergone PCI and were hospitalized to repeat coronary revascularization and elicited their preferences for a new PCI or CABG. Perioperative death, long-term death, myocardial infarction, and repeat revascularization were used to design scenarios describing hypothetical treatments that were labeled as PCI or CABG. PCI was always presented as the option with lower perioperative death risk and a higher necessity to repeat procedure. A conditional logit model was used to analyze patients' choices using R software. A p value < 0.05 was considered statistically significant.

**Results:**

A total of 144 patients participated, most of them (73.7%) preferred CABG over PCI (p < 0.001). The regression coefficients were statistically significant for PCI label, PCI long-term death, CABG perioperative death, CABG long-term death and repeat CABG. The PCI label was the most important parameter (p < 0.05).

**Conclusion:**

Most patients who face the necessity to repeat coronary revascularization reject a new PCI, considering realistic levels of risks and benefits. Incorporating patients' preferences into benefit-risk calculation and treatment recommendations could enhance patient-centered care.

## Introduction

Coronary heart disease is the leading cause of mortality and disability worldwide, responsible for about one-third of all deaths in people over 35 years of age.^1^ There are two revascularization options: percutaneous coronary intervention (PCI) and coronary artery bypass grafting (CABG). Besides the necessity of an open chest surgery for CABG, some crucial distinctions between these treatments are the perioperative risk of death, higher with CABG and the necessity to repeat revascularization, higher with PCI.^2^ Recently, the use of drug–eluting stents have reduced the necessity to repeat revascularization, but the dilemma of the best revascularization strategy is still unanswered.^3^^,^^4^ Therefore, the choice of optimal revascularization strategy is challenging and relies on many factors, such as the number, severity, and position of the narrowed or blocked arteries, patients' overall health, and their preferences for related endpoints, such as recovery time, short-time complications, the necessity to repeat revascularization and long-time survival.^5^

Health care providers have been trying to integrate patients more actively as partners in decisions and the provider must have the skills to involve patients in decision making.^6^ Simply asking patients to rate treatment-related endpoints generally yield no substantial information since they will probably state that they want all the benefits (lower risks for all endpoints). Instead, choice experiments like discrete choice experiments (DCE) force patients to make a trade-off between realistic options, for instance, the option with the lower perioperative death (PCI) versus the option with lower risk to repeat revascularization (CABG).

DCE are frequently used to elicit preferences in a wide range of situations and became the most frequently applied approach in health care.^7^ In a DCE, subjects are presented with a sequence of hypothetical scenarios and are asked to choose between competing alternatives that vary along several characteristics (attributes).

The DCE methodology is grounded in a random utility maximisation (RUM) framework, where the basic assumptions are: 1) any commodity, in this case treatment option (PCI and CABG) can be characterized by key attributes (eg, risk of perioperative death, risk to repeat revascularization) and their levels (e.g., 2%, 35%) and 2) whenever individuals have options to choose from (eg, PCI versus CABG), they make their choice for the option with the greatest utility, which is defined by comparing those attributes' levels.^8^ Utility is a term used by economists to describe the measurement of “usefulness” and “desirability” that a consumer obtains from any good and represents the capacity of a commodity to give satisfaction.

In a recently published systematic review, our research team searched for studies that evaluated stated preferences between PCI and CABG. We identified a shortage of studies that addressed this theme and a lack of standardized methods for evaluating patients' preferences. Even so, fourteen endpoints used to compare PCI and CABG could be identified: atrial fibrillation, heart failure, incision scar, length of stay, long-term death, myocardial infarction, perioperative death, postoperative infection, postprocedural angina, pseudoaneurysm, renal failure, repeat CABG, repeat PCI, and stroke.^9^

Among those who had already undergone PCI, there is no study that evaluated patients' preferences between undergoing a new PCI or CABG, in case new revascularization is indicated. Therefore, this study aimed to provide insights regarding patients' preferences for PCI or CABG in the perspective of hospitalized patients who had to repeat revascularization.

## Methods

### Design

A DCE was developed and administered to a sample of hospitalized patients through individual and face-to-face interviews, from November 2017 to April 2018. The patients were randomly recruited based on their ward number using a list of random numbers at the Instituto Nacional de Cardiologia, a Brazilian tertiary public hospital specializing in cardiology. Patients 18 years old or over were deemed eligible if they had undergone previous PCI and were hospitalized due to coronary disease requiring new revascularization.

Patients who considered themselves unable to understand the experiment were excluded. There were no other exclusion criteria. Ethical approval was obtained from the Instituto Nacional de Cardiologia Ethics Board and written informed consent was obtained from each study participant (CAAE number 63684017.0.0000.5240).

### Discrete Choice Experiment

The DCE was based on endpoints that were identified by the systematic review previously published.^9^ In order to perform the DCE experiment, those endpoints were previously ranked and rated by patients to identify their relative importance. All endpoints were ranked considering a hypothetical scenario. The detailed method used for the patients to rank and rate the endpoints was previously published.^10^ The selection of which attributes should be used in the DCE scenarios is an essential step, since it will only be possible to calculate the trade-offs between the attributes that will be used. We included only four attributes, since the use of all 14 attributes identified in the systematic review would make respondents tired or to use heuristics, a mental shortcut that allows people to make judgments quickly albeit leads to biased preference measures.^11^ The four attributes chose to compose the DCE scenarios were selected considering: 1) long-term death should be included as the reference for marginal rates of substitution; 2) being the most relevant attributes accordingly to patients ranking, and 3) having a significant difference in incidence between PCI and CABG. The four attributes selected were: perioperative death, long-term death, myocardial infarction and repeat revascularization.

In order to use DCE in hospitalized patients, we used visual aids that were specifically developed for this project, in order to include patients with different socioeducational background.^12^ Visual aids improve risk understanding and allow patients to consider themselves able to understand and participate in decisions with answers consistent with economic theory, choosing the alternatives with higher utility.

An example of a DCE scenario presented in this paper to patients is shown in Figure 1: the first attribute (“perioperative death”) is shown with level 3% for PCI (angioplasty) and 8% for CABG (surgery); the second attribute is “death in 5 years”, 22% risk for PCI and 15% for CABG. Each respondent had to choose between PCI and CABG in 12 different scenarios. All scenarios used were shown with the same four attributes, but with different levels combination according to pre-established values. PCI was always presented as the option with lower perioperative death and a higher necessity to repeat procedure (Table 1).

**Figure 1:** A sample discrete choice experiment choice.

**Table 1:** Attributes and levels selected to describe treatment options in the DCE

Attribute	PCI	CABG
Perioperative death	1% - 2% - 3%	4% - 6% - 8%
Long-term death	8% - 15% - 22%	7% - 11% - 15%
Myocardial infarction	6% - 10% - 14%	3% - 5% - 7%
Repeat revascularization	13% - 24% - 35%	1% - 4% - 7%

CABG: coronary artery bypass grafting; PCI: percutaneous coronary intervention.

### Development of the DCE Survey – Selection of Levels

When describing the treatment options in the DCE tasks, the four risk attributes were operationalized by classifying them into three specific levels. The levels for long-term death, revascularization, and myocardial infarction were derived from recent studies comparing PCI versus CABG^4^^,^^13^^–^^17^ in order to make sure that actual levels of risk would be used. The level for perioperative death was selected based on the mean PCI and CABG perioperative mortality rates (2.21% and 6.23%, respectively), according to the Brazilian National Database years 2016 and 2017 (DATASUS)^18^ and was presented in three levels: 1%, 2% or 3% for PCI, and 4%, 6% and 8% for CABG (Table 1).

### Development of the DCE Survey – Designing the Choice Tasks

The NGene Software^19^ was used to design the scenarios, which corresponded to the mechanism by which hypothetical profiles were presented to respondents for preference elicitation in DCE.^11^ A D-Efficient design with no prior information about patients' preferences was used to generate the choice tasks. The order of the choice tasks was randomized among the participants.

All patients were individually and personally interviewed, choosing one option in 12 different scenarios presented in a paper-based questionnaire.

### Statistical Analysis

A conditional logit model was used to analyse patients' choices using R software. Measurement data were presented as mean± standard deviation (x ± SD). A p value < 0.05 was considered statistically significant.

The four risk attributes entered the model as continuous and linear variables. Once patients' preferences for the risk attributes were estimated, it was possible to compute marginal rates of substitution (MRS). The MRS represented the trade-offs between attributes or how much of one attribute patients were willing to sacrifice to obtain more of another attribute. Due to the linear specification of the model, the MRS simply consisted of the ratio of two estimated coefficients.^20^ We followed this approach to compute Maximum Acceptable Risks (MAR) with a 1% increase in the CABG long-term death as the reference.

## Results

Out of 145 recruited patients, 144 gave written informed consent to participate in the study and considered themselves able to understand the experiment. The mean age was 57.5 ± 11.6 years; 74% were men and most patients were married (56%), with a low level of education and low income (Table 2).

**Table 2:** Baseline socioeconomic conditions and characteristics of respondents

Characteristic	Data (N = 144)
Age, years	57.5 (11.6)
Male sex, number (%)	106 (74%)
Annual income, U$	6,838.59 (10,586.82)*
	Married 81 (56%)
Marital status	Single 35 (24%)
	Other 28 (20%)
	≤ 1 year: 5 (3.0%)
	2 – 5 years: 39 (27%)
Level of education, years of study (%)	6 – 9 years: 31 (22%)
	10 – 12 years: 40 (28%)
	College degree: 29 (20%)
	1 – 98 (68%)
Number of previous PCI	2 – 23 (16%)
	3 or more – 23 (16%)

PCI: percutaneous coronary intervention; continuous data are presented as mean (standard deviation).

* conversion based on http://www4.bcb.gov.br/pec/conversao/conversao.asp (1 U$ = 3.49 R$).

Each respondent answered 12 choice tasks, providing thus a total of 1,728 (i.e., 144 times 12) observations for the analysis. Most patients (73.7%) preferred CABG over PCI (p < 0.001). The results for the estimation of preferences are reported in Table 3.

**Table 3:** Estimated Relative Preference Weights

Parameter	Estimate	Standard error	p value
PCI label	- 1.3226	0.6708	< 0.05
PCI perioperative death	- 0.0421	0.0975	NS
PCI long-term death	- 0.0371	0.0172	< 0.05
PCI myocardial infarction	- 0.0314	0.0165	NS
Repeat PCI	- 0.0005	0.0087	NS
CABG perioperative death	- 0.0956	0.0425	< 0.05
CABG long-term death	- 0.0582	0.0287	< 0.05
CABG myocardial infarction	0.0480	0.0407	NS
Repeat CABG	- 0.0657	0.0253	< 0.05

Log-likelihood = −952.35. CABG: coronary artery bypass grafting; PCI: percutaneous coronary intervention; NS: non-significant.

The regression coefficients were statistically significant at 5% level for PCI label, PCI long-term death, CABG perioperative death, CABG long-term death and repeat CABG. The negative coefficients indicate that patients considered the attributes as something undesirable (more risk is worse than less). Notably, the utility function used in the regression model included an alternative specific constant for PCI label and it was not only statistically significant but also the most important parameter, the one with the greatest negative value, meaning that most patients who had to repeat revascularization rejected PCI regardless of the associated risks presented.

## Discussion

The present study is unique since, as far as we know, it is the first one that evaluated patients' preferences among those who had to undergo repeat revascularization after PCI and provides important insights, such as the evidence of a significant variation in the perceived utility of treatments and the noteworthy overall preference for the most invasive option (CABG).

There are few studies that used DCEs as a tool to elicit preferences for coronary revascularization. Our systematic review identified that most studies (83%) used ranking or rating as the method to identify patients' preferences and only two studies (33%)^21^^,^^22^ used hypothetical scenarios. Hornberger et al.^22^ studied a nationwide sample of respondents in a conjoint analysis study considering incision scar, pain, recovery time, days in hospital and repeated treatment. It is noteworthy that the participants considered that PCI would overcome CABG only if the 3-year risk of redoing revascularization declined to less than 28%. Kipp et al.^21^ using a mixed logistic regression analysis, identified that for nearly all quoted risks, patients preferred PCI over CABG, even when the risk of death was double the risk with CABG or the risk of repeat procedures was more than three times that for CABG.

In contrast with the Kipp study, the majority of the patients (73.8%) in this study chose the most invasive option: CABG. This difference may be related to the different population since we considered only patients who had a past history of PCI. Besides that, we must consider some differences in the studies designs. While Kipp et al.^21^ study was based on a threefold risk to repeat PCI over the risk to repeat CABG, with levels between 2 and 5%, we considered CABG risk between 1% and 7% and PCI risk between 13% and 35%. This high risk to repeat PCI was observed in diabetic patients in the Syntax trial,^23^ where 35.3% of patients followed for 5 years had to undergo a new revascularization procedure.

Another important point raised by our findings is that different endpoints are seen differently by patients. However, guidelines' recommendations are based on the use of composite endpoints such as major adverse cardiovascular events (MACE). Endpoints such as death, stroke, myocardial infarction and repeat revascularization are frequently grouped as an attempt to capture the overall treatment effect and the main advantages are the reduction of the duration, sample size and costs of a clinical trial.^24^ The use of MACE assumes that all its components are of equal clinical severity and patients and physicians have a similar perception of each component, assumptions that were false both in our study and in others.^24^^–^^26^ Patients and physicians have distinct perspectives and none of them considered all clinical endpoints equally. The appropriate weight of each component of a composite endpoint would provide a more refined interpretation of the trial data.

An important decision in the application of DCE is whether to present the choices in a labeled or unlabeled form. We decided to adopt labeled scenarios, that is, patients chose between PCI and CABG, and not between option “A” versus “B”. Unlabeled DCEs would be more suitable to investigate trade-offs between attributes, while labeled DCEs may be more suitable to explain real-life choices. Labeled choice sets are considered less abstract and may increase the validity of the results, which may be better suitable to support decision-making at the policy level. The disadvantage is that labels may reduce the attention respondents give to the attributes and some patients may have chosen one option irrespective of their risks.^27^ In our sample, each respondent answered an additional DCE validity test choice task at the end of the DCE section, a dominated question, where PCI represented the treatment with clearly dominant or better attribute levels, i.e., the less invasive option associated with the lesser risks of dying, having a myocardial infarction or repeat treatment. Respondents were expected to choose PCI, but 54 (37.5%) patients chose CABG, which may configure previous PCI rejection and the impact of the label utilization.

### Strengths and Clinical Implications

There are just a few studies regarding patients' preferences between PCI and CABG and this is the first one to analyze patients' preferences specifically for repeated revascularization procedures.

Another strength is the selection of participants, composed of hospitalized patients, waiting for new revascularization. Currently, most health state value sets are obtained from members of the general public, who attempt to imagine what the state would be like, mainly argued for on the basis that the general population are the payers of healthcare. However, patients understand better the consequences of their choices and what it is like to live with that health condition. This minimizes one of the major concerns with DCEs that is the hypothetical bias related to patients' disinterest or inattention towards hypothetical scenarios, while patients facing the health problem would be more involved with the experiment.

Current cardiology guidelines may benefit from including patients' preferences into their recommendations. For instance, taking into consideration the results for patients with three-vessel disease of the Syntax trial, the 11.4% long-term mortality in the CABG group (coefficient value – 0.0582) would be equivalent to 17.9% ((-0.0582/-0.0371)*11.4) long-term mortality in PCI group (coefficient value −0.0371). Based on the value of the parameters identified in our regression model, even with the higher PCI long-term mortality (13.9%), this 2.5% long-term mortality difference, shown in the Syntax trial, would not be sufficient to influence patients' preferences in favour of CABG.

### Limitations

The results of our study are limited by the use of a small sample size from a single tertiary hospital, which may limit the generalizability of our results.

There may be some interaction effects, since patients may have valued particular attributes or levels differently because of their previous particular experience. Another issue is that the attributes were modelled as continuous variables to make it easier to understand and we considered the effect of levels preferences as linear, which may not be realistic since the value of changing from low to moderate risk not necessarily is the same value of changing risk from moderate to severe.

## Conclusion

Despite the important trade-offs between PCI and CABG, such as the necessity to repeat revascularization, patients' preferences have been poorly explored. In a DCE with a sample of hospitalized patients with coronary disease and previous PCI, our results support that most patients reject a new PCI and prefer CABG when facing realistic risk levels of each option.
